# Rapidly Progressive Bilateral Syphilitic Optic Neuritis in an Elderly Patient: A Case Report

**DOI:** 10.7759/cureus.97886

**Published:** 2025-11-26

**Authors:** Masahiko Funatsu, Makiko Wakuta, Ayano Sakuma, Waka Hamada, Junki Sunada, Fumiaki Higashijima, Yuki Wasai, Kazuhiro Kimura

**Affiliations:** 1 Ophthalmology, Yamaguchi University, Ube, JPN

**Keywords:** bilateral vision loss, elderly patient, syphilitic optic neuritis, syphilitic uveitis, treponema pallidum

## Abstract

Syphilitic optic neuritis (SON), caused by Treponema pallidum infection, typically demonstrates favorable visual outcomes when promptly diagnosed and appropriately treated with high-dose intravenous penicillin therapy. However, certain patient factors including advanced age, severe initial visual impairment, and rapid disease progression may influence treatment outcomes. This case report aims to highlight the clinical challenges and poor prognostic factors associated with SON in elderly patients by presenting a rare case of rapidly progressive bilateral visual dysfunction despite optimal medical management. To achieve this objective, we present a detailed case analysis involving comprehensive ophthalmologic examination, laboratory investigations, and treatment response evaluation in an 80-year-old woman who developed acute bilateral vision loss. This study presents a comprehensive case report involving the systematic documentation, clinical analysis, and treatment outcomes of bilateral SON with neuroretinitis manifestation selected based on the unusual demographic presentation and a typical treatment response. Significant and concerning findings indicate that despite early diagnosis confirmed by positive serological tests (RPR 2.9 R.U., TPHA 6.24 R.U.) and prompt initiation of high-dose intravenous benzylpenicillin potassium therapy (18 million units daily for 14 days), visual function recovery was minimal, with persistent severe visual impairment in both eyes at one-month follow-up. However, inflammatory signs including optic disc swelling and peripapillary exudates showed marked improvement, though RPR titers remained persistently low-positive. Advanced therapeutic interventions including sub-Tenon triamcinolone acetonide injections and oral amoxicillin supplementation were employed because RPR titers had not turned negative, and vision improvement had not been achieved. Our case demonstrates that SON in elderly patients may exhibit poor visual prognosis despite appropriate treatment protocols. Hence, patient age and initial disease severity are significant prognostic factors, and, unquestionably, this represents an important clinical consideration for ophthalmologists managing ocular syphilis. For this reason, early recognition and aggressive treatment initiation remain crucial, though visual recovery expectations should be tempered in elderly patients with severe initial presentations.

## Introduction

Syphilis, caused by Treponema pallidum, remains one of the most significant sexually transmitted infections worldwide, presenting with diverse clinical manifestations across multiple organ systems [1]. The incidence of syphilis has been rapidly increasing in developed countries, including the United States, Canada, and the United Kingdom [2,3]. In Japan, this trend has been particularly pronounced since 2021, with 13,258 cases reported in 2022 alone [4].

Ocular manifestations of syphilis can occur at any stage of the disease and include scleritis, iritis, retinochoroiditis, retinal vasculitis, and optic neuritis. Ocular syphilis is treated as neurosyphilis regardless of CSF findings, and syphilitic optic neuritis (SON) is considered a form of neurosyphilis and requires aggressive treatment with high-dose intravenous penicillin G therapy from the early stages of disease [5]. When promptly diagnosed and appropriately treated, SON generally carries a relatively favorable prognosis with good visual recovery [6].

However, certain patient factors may influence treatment outcomes. Age-related factors, severity of initial visual impairment, and duration of symptoms before treatment initiation have been identified as potential prognostic indicators. We report an unusual case of late-onset bilateral SON in an 80-year-old woman who experienced poor visual recovery despite early diagnosis and appropriate high-dose penicillin therapy, with persistently positive RPR titers.

## Case presentation

**Figure 1:**
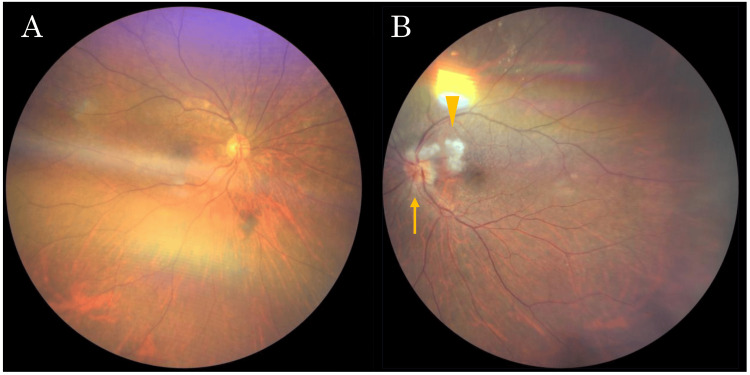
Fundus photograph of the patient at the time of initial examination. There were no obvious findings in the right eye (A), while erythematous swelling of the optic disc (arrow) and peripapillary cotton-wool spots (arrowhead) in the left eye (B).

The patient was an 80-year-old woman. She had decreased vision in her left eye for 2 - 3 days and initially consulted a local ophthalmologist. She was diagnosed with optic papillitis as her left eye visual acuity had deteriorated to hand motion and the optic disc demonstrated swelling with blurred margins. The following day, she developed vision loss in her right eye and was referred to Yamaguchi University Hospital for comprehensive evaluation and treatment. The patient had no significant past medical history of ophthalmologic or systemic diseases, and no family history of similar conditions. Visual acuity from the day before was 20/20 in the right eye and hand motion in the left eye. On presentation, visual acuity was 20/300 in the right eye and counting fingers at 50 cm in the left eye. The direct light reflections were diminished in both eyes. Additionally, relative afferent pupillary defect was positive in the left eye. Intraocular pressure measurements were 12 mmHg in the right eye and 15 mmHg in the left eye. Critical flicker fusion frequency revealed 19 Hz in the right eye, while it was unmeasurable in the left eye. Slit-lamp biomicroscopy demonstrated fine keratic precipitates and 2+ inflammatory cells in the anterior chamber of the right eye and 1+ in the left eye. Slit-lamp microscopy revealed a grade 3 cataract in the right eye according to the Emery-Little classification, and the left eye was pseudophakic. The right eye had good visual acuity of 20/20, and although it had cataracts, they did not affect vision. The left eye, which had poor vision, was pseudophakic due to a previous cataract surgery. Therefore, it is believed the cataract in the left eye was not related to this episode. The right eye showed no abnormal findings at this initial assessment (Figure 1A), whereas fundoscopic examination of the left eye revealed erythematous optic disc swelling with peripapillary cotton-wool spots (Figure 1B).

**Figure 2:**
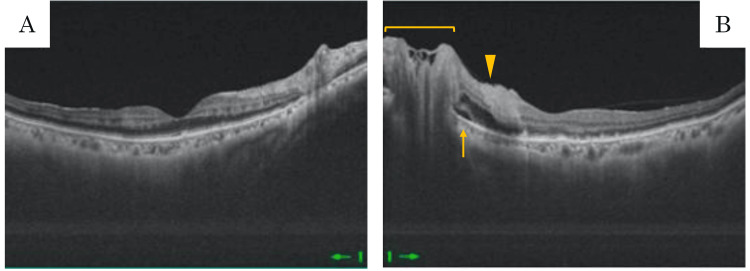
OCT images of the patient at the time of initial examination. The ellipsoid zone line appeared indistinct and showed focal disruptions in the right eye (A) and left eye (B). There also seemed to be deposits in the RPE layer. Swelling of the optic disc (bracket), peripapillary retinal edema (arrowhead), and serous retinal detachment (arrow) in the left eye. OCT: Optical coherence tomography; RPE: retinal pigment epithelium

Neither eye demonstrated vitreous opacity or macular edema. Swept-source optical coherence tomography (OCT) revealed an indistinct ellipsoid line with localized breaks in both eyes. Deposits were also observed in the retinal pigment epithelium (RPE) layer. In the left eye, optic disc swelling, peripapillary retinal edema, and serous retinal detachment were noted (Figures 2A, 2B)

**Figure 3:**
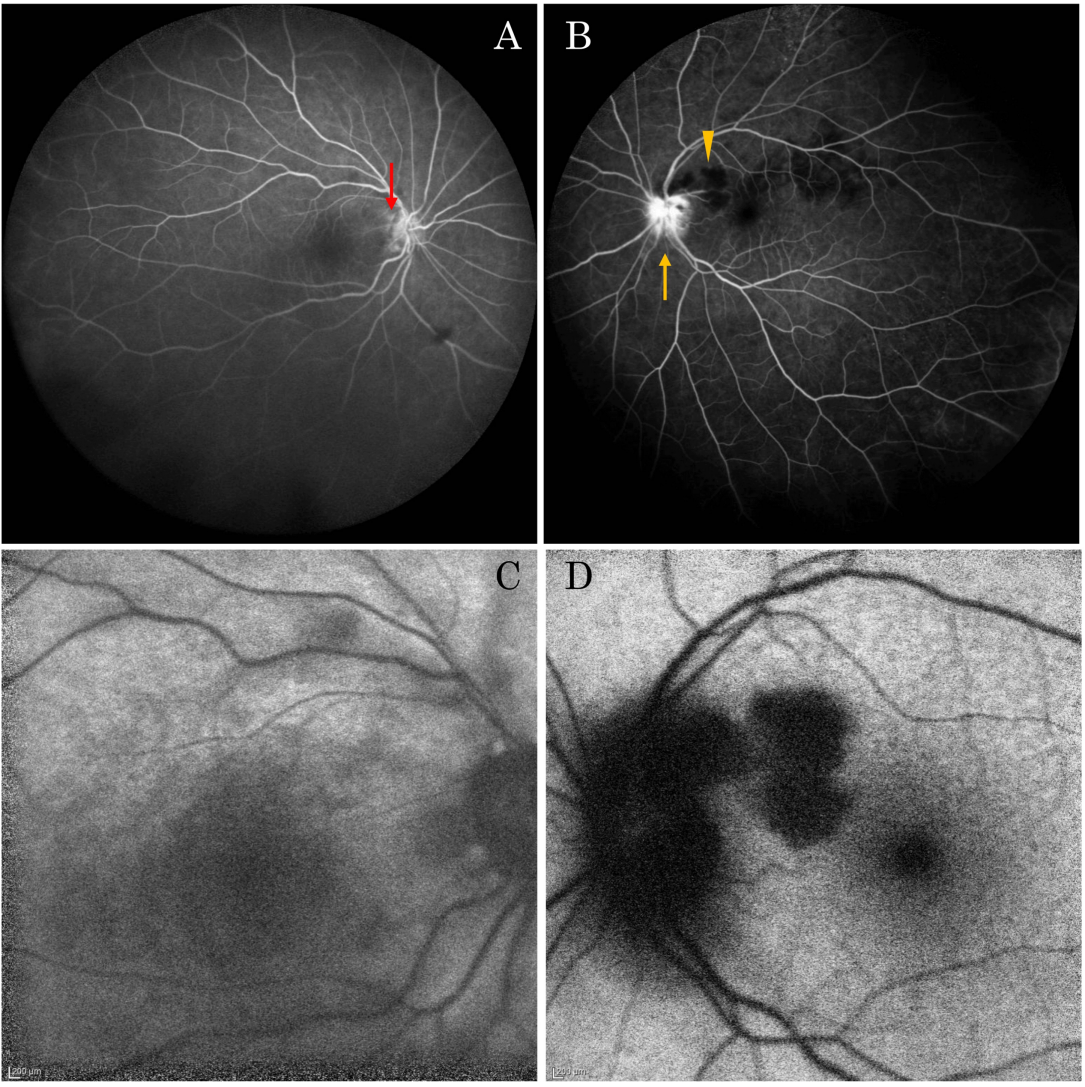
Fluorescein angiography and autofluorescence fundus photography images of the patient at the time of initial examination. Hyperfluorescence due to the conus was observed on the temporal side of the optic disc in the right eye (A) (red arrow), and the optic disc leakage (yellow arrow) and hypofluorescence (arrowhead) due to cotton-wool spots in the left eye (B). The right eye (C) and the left eye (D) showed hypofluorescence on fundus autofluorescence imaging.

Fluorescein angiography revealed optic disc leakage and hypofluorescence corresponding to the soft exudates in the left eye, while the right eye remained normal (Figures 3A, 3B).

**Figure 4:**
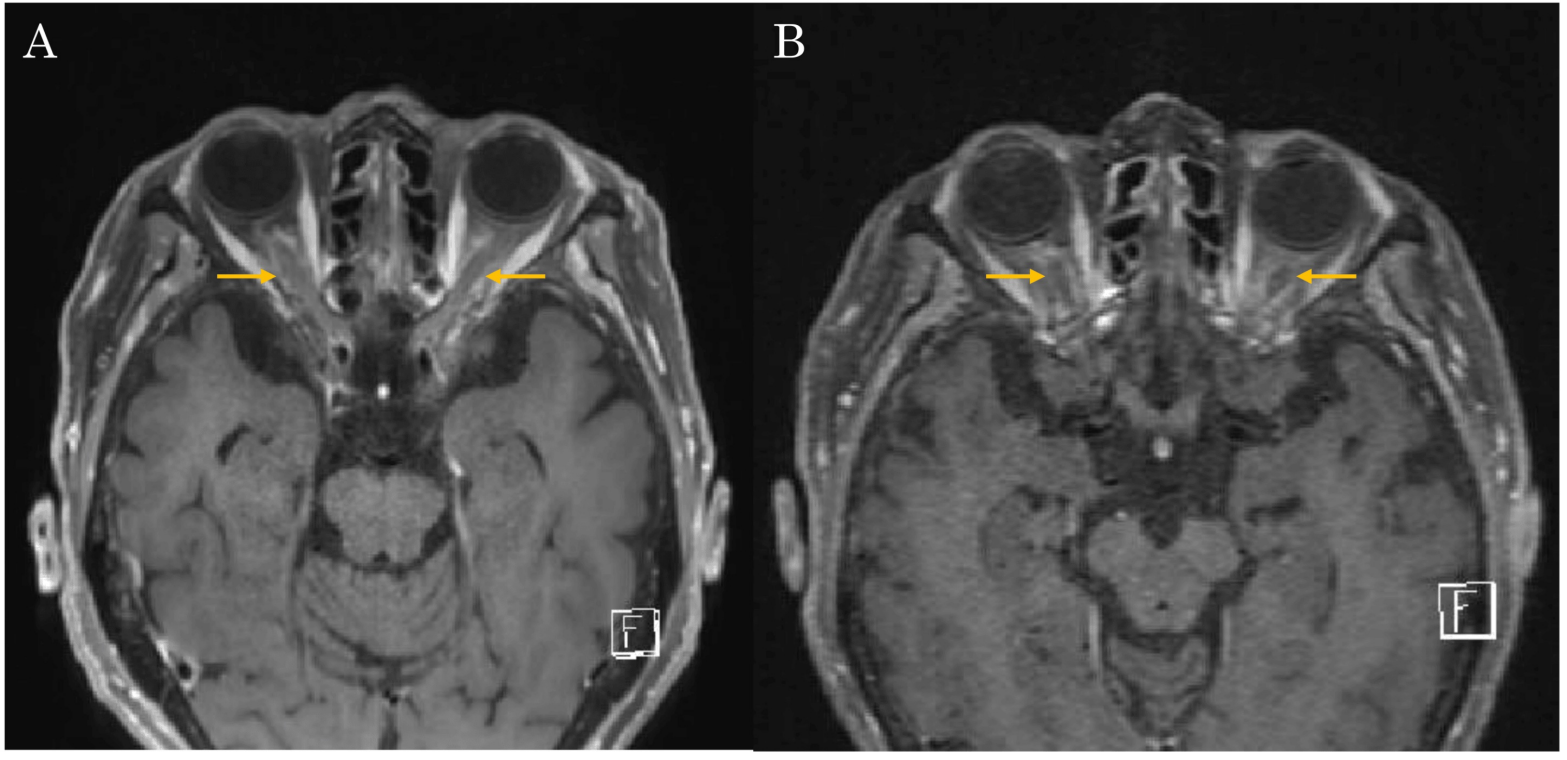
Contrast-enhanced MRI images of the orbits before and after antisyphilitic therapy. Enhancing effects were observed in the optic nerves (arrow) of both eyes (A). After one month of treatment, the high signal in the optic nerves of both eyes was slightly reduced (B).

Contrast-enhanced magnetic resonance imaging of the orbits showed enhancement of both optic nerves (Figure 4A).

**Figure 5:**
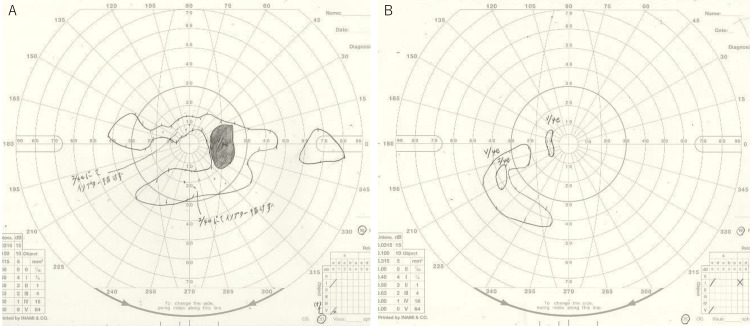
Goldmann visual field test before antisyphilitic therapy of the case. Before the start of treatment, central dark spots and extensive visual field constriction were observed in both eyes at the time of initial examination: (A) right eye and (B) left eye.

Laboratory investigations revealed positive syphilis serologies with rapid plasma reagin (RPR) of 2.9 R.U. (normal <1.0 R.U.) and Treponema pallidum hemagglutination assay (TPHA) of 6.24 R.U. (normal <1.0 R.U.). On the day following the initial presentation, the right eye developed erythematous optic nerve swelling similar to the left eye. Goldmann visual field testing demonstrated central scotomas and severe peripheral visual field constriction in both eyes (Figures 5A, 5B).

**Figure 6:**
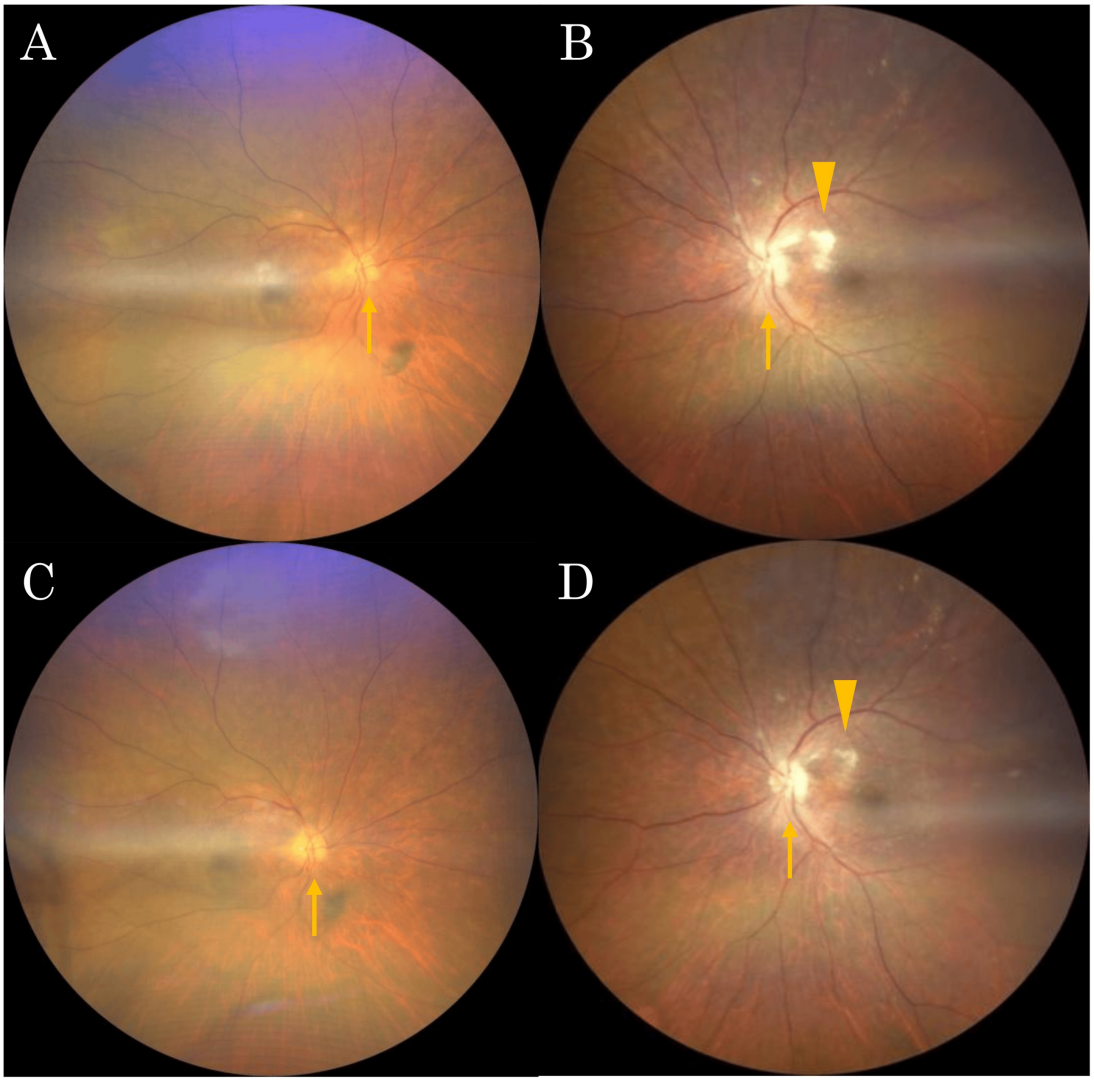
Fundus photographs of both eyes after antisyphilitic therapy in the case. After intravenous infusion, erythematous swelling of the optic disc (arrow) and peripapillary soft exudates (arrowhead) in both eyes were reduced: (A) right eye and (B) left eye. One month after the start of treatment, fundus findings in both eyes improved further: (C) right eye and (D) left eye.

Based on the positive syphilis serologies combined with optic neuritis (right eye) and optic neuroretinitis (left eye), a diagnosis of ocular syphilis was established. The patient was admitted for treatment on the same day. The apparent route of infection in this case remained unclear. The last sexual intercourse occurred over 50 years ago. There is no history of blood transfusions. While congenital factors are unknown, the patient was born in the 1940s when syphilis was prevalent in Japan, making congenital syphilis a possibility. Furthermore, since the cerebrospinal fluid culture test was negative for syphilis, drug resistance could not be evaluated. The patient also reported numbness in both upper and lower extremities, raising suspicion for neurosyphilis. Consequently, cerebrospinal fluid examination was performed by the neurology department on the day following admission. It was negative for syphilis. High-dose intravenous benzylpenicillin potassium therapy was initiated (3 million units every four hours, totaling 18 million units daily for 14 days) following neurosyphilis treatment protocols. Following commencement of antibiotic therapy, the erythematous optic disc swelling and peripapillary cotton-wool spots showed improvement in both eyes (Figures 6A, 6B), though serological testing revealed a persistently low-positive RPR of 3.5 R.U.

**Figure 7:**
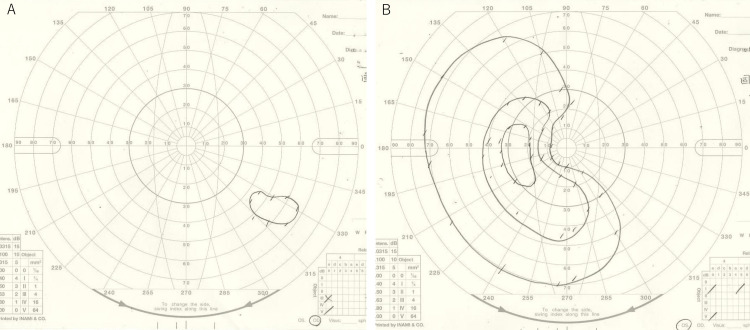
Goldmann visual field test after antisyphilitic therapy of the case. After one month of continued antibiotic treatment, visual field constriction worsened after treatment in the right eye (A). The peripheral visual field in the left eye improved after treatment (B).

On treatment day 13, sub-Tenon triamcinolone acetonide injections were administered to both eyes due to a lack of visual acuity improvement. Systemic corticosteroid therapy was not administered because this patient whose blood test was a positive result for hepatitis C virus antibodies. Despite this intervention, visual function showed no significant recovery. On treatment day 17, oral amoxicillin (250 mg three times daily for 14 days) was added to the regimen due to persistent positive syphilis serology. At 19 days post-treatment initiation, visual acuity remained severely impaired with hand motion at 50 cm in the right eye and 20/40 (uncorrectable) in the left eye. Critical flicker fusion frequency showed no improvement from baseline (19 Hz right eye, unmeasurable left eye). Goldmann visual field testing demonstrated partial improvement in peripheral vision in the left eye but deterioration in the right eye (Figure 7A, 7B).

At one-month follow-up, fundoscopic findings continued to improve in both eyes (Figures 6C, 6D), and repeat contrast-enhanced MRI showed a slight reduction in optic nerve signal enhancement bilaterally (Figure 4B). However, serological testing revealed persistent low-positive RPR at 6.4 R.U., indicating ongoing serological activity despite clinical improvement of inflammatory signs.

## Discussion

Although the exact route of infection in this case remains unclear, the patient was born in Japan during the 1940s, when syphilis was prevalent, raising the possibility of congenital syphilis. This suggests that syphilis infection should be considered even when there is no clear history of infection in elderly patients with uveitis. This case represents an unusual presentation of SON in an 80-year-old woman with rapidly progressive bilateral visual dysfunction and poor treatment response despite early diagnosis and appropriate therapy (Table 1) [7-9].

**Table 1:** Comparison of this case with other cases of syphilitic optic neuritis RPR: Rapid plasma reagin

Lead author	Year of Publication	Age/Gender	Affected eye	RPR before treatment (R.U.)	Visual acuity		Treatment details
					Pre-treatment	Post-treatment	
Kaoruko Yamamoto [7]	2004	46/male	Right	positive	Manual valve in front	20/50	Intravenous benzylpenicillin potassium therapy 24 million units/day, 2 weeks
			Left		20/100	20/40	
Shun Yamashita [8]	2022	69/male	Left	positive	counting fingers at 10 cm	50/100	Intravenous benzylpenicillin potassium therapy 24 million units/day, 2 weeks
								Kyosuke Nonaka [9]	2024	61/female	Left	131.5	20/50	20/50	Oral amoxicillin 1,500 mg/day, 5 weeks
Masahiko Funatsu	2025	80/female	Right	2.9	20/300	hand motion at 50 cm	Intravenous benzylpenicillin potassium therapy 18 million units/day, 2 weeks ＆ Oral amoxicillin 750 mg/day, 2 weeks								
			Left		counting fingers at 50 cm	20/40	

The combination of positive serological tests (RPR 2.9 R.U., TPHA 6.24 R.U.) with characteristic bilateral optic neuritis and neuroretinitis findings confirmed the diagnosis of ocular syphilis, which is increasingly recognized as cases of syphilis continue to rise globally [3,4].

The diagnostic approach in this case followed established protocols for ocular syphilis evaluation. While RPR can yield false-positive results in elderly patients due to various conditions including collagen diseases, liver disease, or malignancy [10], the combination of positive TPHA and compatible clinical presentation strongly supported the diagnosis. The patient's complaint of limb numbness raised suspicion for neurosyphilis; however, cerebrospinal fluid examination revealed no abnormalities. Ideally, CSF-VDRL testing should have been performed to definitively rule out neurosyphilis, representing a limitation in our diagnostic workup. Nevertheless, ocular syphilis is treated as neurosyphilis regardless of CSF findings, making this distinction less critical for treatment decisions [11].

Acute syphilitic posterior placoid chorioretinitis (ASPPC) is a lesion characteristic of syphilis, first named by Gass et al. in 1990 [12]. Fundus findings typically show gray to yellowish-white lesions extending from the fovea to the parapapillary region. OCT images reveal irregularities in the ellipsoid zone, nodular lesions, and thickening of the RPE [13]. The present OCT also showed irregularities in the ellipsoid zone and nodular lesions, raising suspicion of ASPPC. However, the presence of drusen on fundus photography and hypofluorescence on autofluorescence imaging suggests age-related retinal pigment epithelial atrophy rather than ASPPC.

Our treatment approach adhered to current guidelines for neurosyphilis management. High-dose intravenous benzylpenicillin potassium (18 million units daily for 14 days) was administered following CDC recommendations [11], as ocular syphilis is considered equivalent to neurosyphilis. The addition of sub-Tenon triamcinolone acetonide injections on day 13 was based on reports suggesting the potential benefit of adjunctive corticosteroids in severe syphilitic optic neuropathy [5]. Additionally, this case tested positive for hepatitis C virus antibodies, posing a risk of hepatitis with systemic steroid administration. Therefore, we chose local rather than systemic steroid administration. However, evidence regarding the efficacy of steroids for SON remains limited and controversial.

Several factors likely contributed to the poor visual prognosis in this case, contrasting with the generally favorable outcomes reported in the literature [6]. First, the patient's advanced age (80 years) was significantly higher than the typical demographic for ocular syphilis, which predominantly affects individuals aged 20-50 years [14]. This case suggests that aging may be associated with reduced neural recovery capacity and increased susceptibility to irreversible optic nerve damage. Second, the severity of initial visual impairment was substantial, with visual acuity of 20/300 and counting fingers at presentation. Previous systematic reviews have identified poor initial visual acuity as a significant predictor of unfavorable outcomes in syphilitic uveitis, along with prolonged symptom duration and presence of optic neuropathy [15]. Third, despite treatment initiation within days of symptom onset, the rapid inflammatory process may have already caused irreversible structural damage to the optic nerves before effective antimicrobial levels were achieved. Fourth, although cerebrospinal fluid culture results were negative in this case, the causative pathogen may have been a drug-resistant bacterium. While drug resistance in Treponema pallidum has been a concern in recent years, previous reports primarily indicate resistance to macrolide antibiotics, and no resistance to penicillin has been reported [16]. Therefore, this possibility is considered low.

The persistence of low-positive RPR titers (remaining at 6.4 R.U. at one month) despite appropriate antibiotic therapy warrants discussion. Delayed serological response or persistent low-level positivity can occur in cases of late-stage syphilis or in elderly patients [7]. Given the patient's birth before World War II, when syphilis prevalence was extremely high in Japan, the possibility of remote infection with late manifestation cannot be excluded. However, the absence of classic signs of congenital syphilis, such as Hutchinson's triad (interstitial keratitis, eighth nerve deafness, and Hutchinson's teeth), argues against this diagnosis. Close serological monitoring remains essential to ensure eventual conversion to negative or stable low-positive titers, though some patients may maintain persistently low-positive results following successful treatment.

This case underscores important clinical implications for ophthalmologists managing ocular syphilis. While SON generally carries a favorable prognosis with prompt diagnosis and treatment [6], patient-specific factors including advanced age and severe initial visual impairment may significantly influence outcomes. The increasing incidence of syphilis in developed countries, including Japan where cases have risen dramatically since 2021 [4], necessitates heightened clinical awareness across all age groups. Early recognition and aggressive treatment initiation remain the cornerstone of management, though realistic prognostic expectations should be communicated to elderly patients presenting with severe visual dysfunction. Future research should focus on identifying additional prognostic factors and potentially exploring adjunctive therapies to optimize visual outcomes in high-risk patients.

## Conclusions

The incidence of syphilis continues to rise globally. This case demonstrates that despite rapid diagnosis within days of symptom onset and prompt initiation of appropriate high-dose intravenous penicillin therapy, visual outcomes may remain poor in certain patient populations. The combination of advanced patient age, severe initial visual impairment, and rapid disease progression appears to contribute to unfavorable prognosis even with optimal medical management. While early diagnosis and immediate treatment initiation remain essential for maximizing visual recovery in ocular syphilis, realistic prognostic expectations should be discussed with elderly patients presenting with severe visual dysfunction. Holistic care involving multidisciplinary follow-up (neurology, infectious diseases, ophthalmology) is considered necessary. This case highlights that patient-specific factors may significantly influence treatment outcomes despite adherence to established therapeutic protocols, emphasizing the need for individualized patient counseling regarding visual prognosis. Further case studies on late-onset SON are considered necessary.
